# The Wnt-target gene *Dlk-1* is regulated by the Prmt5-associated factor Copr5 during adipogenic conversion

**DOI:** 10.1242/bio.201411247

**Published:** 2015-02-13

**Authors:** Conception Paul, Claude Sardet, Eric Fabbrizio

**Affiliations:** 1 Equipe labellisée Ligue Contre le Cancer, Institut de Génétique Moléculaire de Montpellier, CNRS, UMR5535, 34293 Montpellier, France; 2 Université Montpellier I and II, 34000 Montpellier, France; 3 Institut de Recherche en Cancérologie de Montpellier, Inserm, U1194, 34298 Montpellier, France

**Keywords:** β-catenin, Copr5, Dlk-1, Prmt5, Adipocyte, Differentiation

## Abstract

Protein arginine methyl transferase 5 (Prmt5) regulates various differentiation processes, including adipogenesis. Here, we investigated adipogenic conversion in cells and mice in which *Copr5*, a Prmt5- and histone-binding protein, was genetically invalidated. Compared to control littermates, the retroperitoneal white adipose tissue (WAT) of *Copr5* KO mice was slightly but significantly reduced between 8 and 16 week/old and contained fewer and larger adipocytes. Moreover, the adipogenic conversion of *Copr5* KO embryoid bodies (EB) and of primary embryo fibroblasts (Mefs) was markedly delayed. Differential transcriptomic analysis identified Copr5 as a negative regulator of the *Dlk-1* gene, a Wnt target gene involved in the control of adipocyte progenitors cell fate. *Dlk-1* expression was upregulated in *Copr5* KO Mefs and the Vascular Stromal Fraction (VSF) of *Copr5* KO WAT. Chromatin immunoprecipitation (ChIP) show that the ablation of Copr5 has impaired both the recruitment of Prmt5 and β-catenin at the *Dlk-1* promoter. Overall, our data suggest that Copr5 is involved in the transcriptional control exerted by the Wnt pathway on early steps of adipogenesis.

## INTRODUCTION

Adipose tissue has various regulatory functions in the metabolism of animals and acts both as a fat reservoir and an endocrine/paracrine/autocrine organ that can expand throughout the entire lifespan. This functional plasticity can lead to pre-adipocyte hyperplasia and adipocyte hypertrophy. Fat tissue includes many different cellular components, including preadipocytes, multipotent stem cells (MSC) and mature adipocytes (Zeve et al., 2009).

Adipogenesis is a multi-step process during which the increase in adipocyte number is triggered by various extra- and intra-cellular signalling factors that induce MSC conversion into preadipocytes (Tang and Lane, 2012). This commitment is restricted to the adipocyte lineage upon activation of a transcriptional programme in which key factors of adipocyte differentiation like C/Ebpα and Pparγ are induced (MacDougald and Lane, 1995; Rosen and MacDougald, 2006; Tontonoz and Spiegelman, 2008). Among sensors of external signals to trigger adipocyte differentiation during embryonic development and adult life, the Wnt signalling pathway is crucial for progenitor fate determination and acts through Dlk-1 that regulates negatively preadipocyte proliferation (Moon et al., 2002; Mortensen et al., 2012; Smas and Sul, 1993).

In association with protein complexes involved in different phases of transcription, Protein arginine methyl transferase 5 (Prmt5) was implicated in myogenic, adipogenic and glial cell differentiation (Dacwag et al., 2009; Huang et al., 2011; LeBlanc et al., 2012; Paul et al., 2012). Consistently with this biological function, we reported previously that the depletion of the Prmt5- and histone-associated protein Copr5 delays the myogenic conversion (Paul et al., 2012), suggesting that Copr5 elicits a fine tuning of Prmt5 functions related to cell differentiation.

In this work, we generated mice in which *Copr5* was genetically invalidated and show that adipogenic conversion was delayed *in vitro* both in EBs and Mefs derived from these mice compared to control cells. In addition, the retroperitoneal WAT of *Copr5* KO (KO) mice was slightly reduced and contained larger adipocytes compared to control mice. Finally, we show that the expression of *Dlk-1* was upregulated in KO cells and coincides with an altered recruitment of Prmt5 and β-catenin to the *Dlk-1* promoter. Altogether, our data highlight unsuspected functions of Copr5 in the modulation of adipogenic differentiation, notably through an impact on the Wnt/β-catenin-dependent regulation of the *Dlk-1* promoter.

## RESULTS AND DISCUSSION

### Adipogenesis is impaired in *Copr5* KO cells

**Fig. 1.**
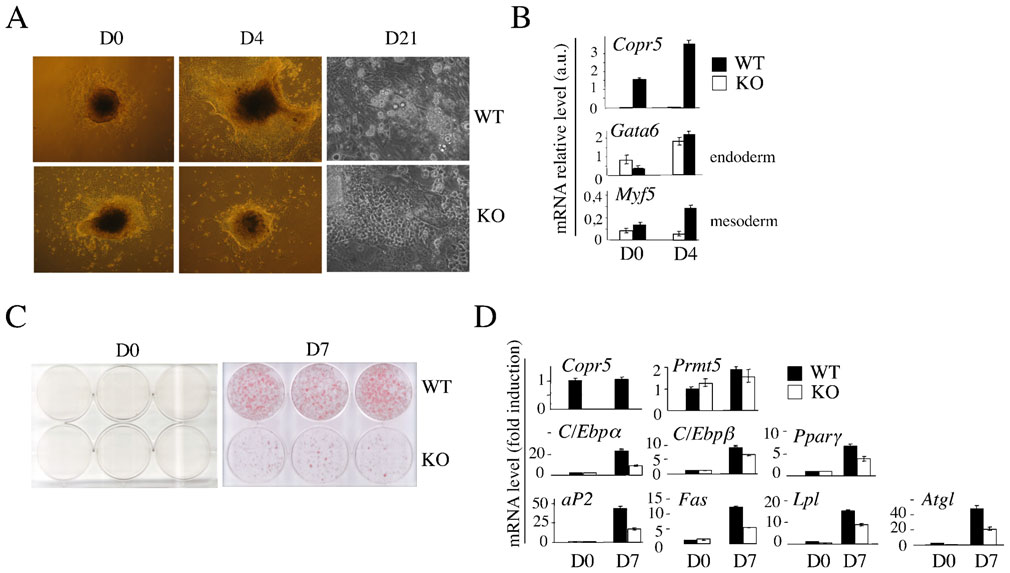
Adipogenic conversion is delayed in *Copr5* KO cells. (A) Phase contrast micrographs of EB generated from WT and KO ES cells at D0 (third day of treatment with 10^−6^ M retinoic acid) and at D4 and D21 after induction of EB adipogenic differentiation with insulin and triiodothyronin (T3). (B) RNA was extracted at D0 and D4 from WT and KO EB and used in RT-qPCR analysis to assess the expression profile of the indicated markers. Normalisation was done with *S26* RNA and values are expressed in arbitrary units (a.u.). (C) O Red Oil staining of post-confluent (D0) and differentiating (D7) WT and KO Mefs. Differentiation was induced at D0 in the presence of insulin and rosiglitazone. (D) mRNA expression in differentiating WT and KO Mefs was monitored by RT-qPCR and is shown at D0 and D7. Normalisation was done with S26 RNA. Values are expressed as the fold change compared to control and are the mean±s.e.m. of three independent experiments.

We generated a mouse model in which the *Copr5* gene was genetically invalidated by homologous recombination (supplementary material Fig. S1). In contrast to *Prmt5* loss of function, which is early embryonic-lethal due to loss of pluripotent cells (Tee et al., 2010), *Copr5* KO mice were viable and ES cells could be derived from KO blastocysts, indicating that the Copr5-independent functions of Prmt5 are not essential for mouse development. However, when tested for their capacity to differentiate *in vitro* into adipocytes (Dani et al., 1997), lipid droplets were observed mostly in WT EBs cultures at D21 (Fig. 1A). Moreover, the mRNA level of *Myf5*, which was used as a read-out of differentiation, confirmed that mesodermic lineage differentiation was already delayed at D4 in KO compared to WT EBs (Fig. 1B). O Red Oil staining and mRNA analysis showed that adipogenic conversion was also very ineffective in KO compared to WT Mefs (Fig. 1C,D), as well as in *Copr5* shRNA-treated F-442A preadipocyte cell line (supplementary material Fig. S2D,E). Altogether, these data indicate that Copr5 is required for an efficient adipogenic conversion of cells in culture. Although the mRNA level of *Copr5* did not vary significantly during fat tissue development (supplementary material Fig. S2A) (Birsoy et al., 2011), it was induced at the early time points of the adipogenic conversion of WT Mefs, preceding those of transiently-expressed players involved in the initiation of adipocyte differentiation, including *Krox20*, *Klf4* and *Klf5* (Birsoy et al., 2011; Chen et al., 2005). As expected, the mRNA level of these factors was downregulated in KO Mefs (supplementary material Fig. S2B). Surprisingly, a transient ectopic re-expression of Copr5 in KO cells failed to rescue their capacity to differentiate (supplementary material Fig. S2C). These results suggest that Copr5 deficiency had impacted on very early and irreversible events required for the adipogenic conversion of Mefs.

### Copr5 controls the expression of *Dlk-1* gene, a key regulator of preadipocyte differentiation

**Fig. 2.**
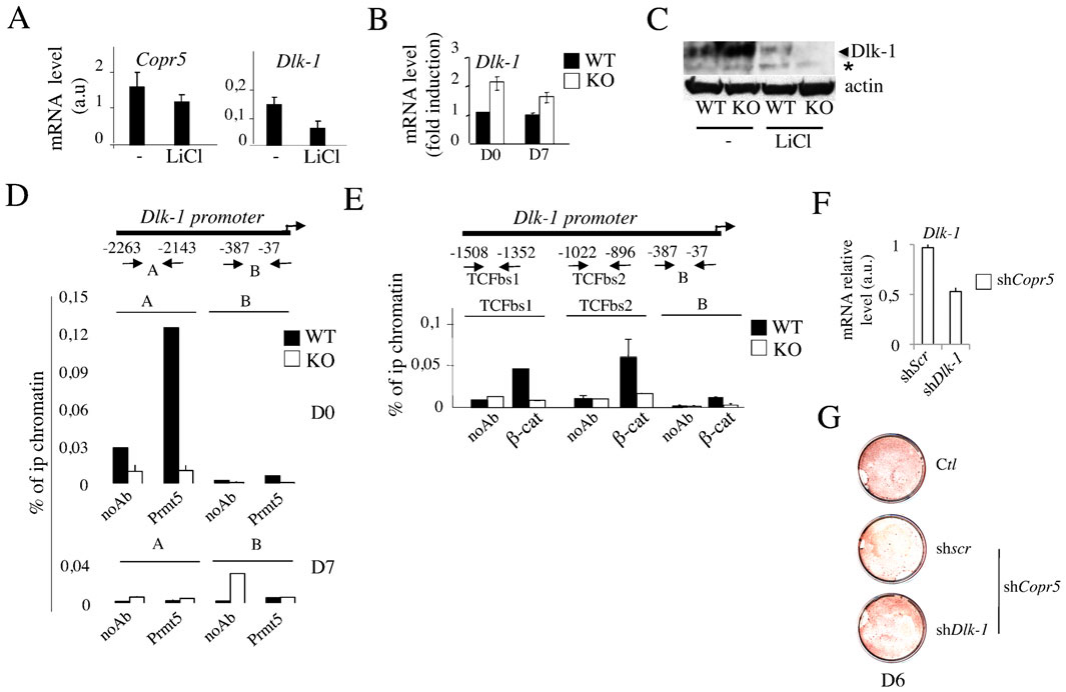
*Dlk-1* upregulation in *Copr5* KO Mefs is related to impaired recruitment of Prmt5 to the *Dlk-1* promoter. (A) Expression of indicated mRNAs in Mefs was monitored by RT-qPCR after treatment of the cells with LiCl (10 mM) for 24 h. Results are expressed in arbitrary units (a.u.). (B) Expression of *Dlk-1* mRNA in WT and KO Mefs was monitored by RT-qPCR at D0 and D7 of differentiation. Normalisation and expression was done as in Fig. 1D. (C) Western Blot detection of WT and KO Mef whole cell extracts treated as in (A) using an anti-Dlk-1 antibody is shown. (D) Prmt5 recruitment to A and B regions of the *Dlk-1* promoter in WT and KO Mefs was analysed by ChIP at D0 and D7. Values are expressed as the percentage of immunoprecipitated (ip) chromatin relative to input and are the mean±s.e.m. of triplicates. No antibody (noAb) was used as negative control. (E) Immunoprecipitation was performed as in (D) to assess the recruitment of activated β-catenin (β-cat) to three regions of the *Dlk-1* promoter: two of them encompass the TCF binding sites 1 and 2 (TCFbs1 and TCFbs2), the third one is B, as in (D). (F) Expression of *Dlk-1* mRNA in sh*Copr5* F442A-treated cells was monitored by RT-qPCR upon inactivation of *Dlk-1* using sh*RNA* (sh*Dlk-1*) encoding retroviral particles. A scramble sh*RNA* (sh*Scr*) was used as control. Normalisation and expression were performed as in (B). (G) O Red Oil staining at D6 of sh*Copr5* F442A-treated cells infected as in (F). *: non specific band.

To unravel the molecular mechanisms that could explain the poor capacity of KO Mefs to undergo an adipogenic conversion, we compared their transcriptome profile with that of WT Mefs (supplementary material Table S1). Notably, among the 538 genes that were significantly deregulated (Zr>2;Zpval>0.05) in KO cells, 34 were bona fide Wnt/β-catenin target genes (p = 4.67×10^−12^, Fisher's test) (supplementary material Fig. S3A–C). Biochemical fractionation showed that KO Mefs contained higher amounts of the activated form of β-catenin in their nucleus than WT cells (supplementary material Fig. S3D), a difference that was lessened upon treatment with either LiCl or C59, two chemicals known to activate and inhibit the Wnt pathway, respectively (supplementary material Fig. S3D). Consistently, reporter assays confirmed that TCF/β-catenin transcriptional activity was increased in KO cells (supplementary material Fig. S3E). Within this list, we noticed the presence of *Dlk-1*, a gene encoding a key regulator of adipose tissue homeostasis *in vivo* whose expression in WAT is linked to inhibition of adipocyte differentiation (Moon et al., 2002; Mortensen et al., 2012; Smas and Sul, 1993). Interestingly, *Dlk-1* is one of the few non-conventional target genes of the Wnt pathway that were reported to be directly repressed by the TCF/β-catenin complex (Blauwkamp et al., 2008; Weng et al., 2009). Analysis of *Dlk-1* expression confirmed its sensitivity to LiCl in WT Mefs and its upregulation in KO Mefs (Fig. 2A–C), suggesting that this gene was derepressed in KO cells, despite their high levels of activated form of β-catenin. Based on our previous reports showing that Copr5/Prmt5 complex could be involved in transcriptional repression (Lacroix et al., 2008), we hypothesised it could be involved in the repression of the *Dlk1* promoter. Consistently, ChIP performed in Mefs during the early phase of their adipogenic conversion showed that Prmt5 was present on the *Dlk-1* promoter in WT but not KO Mefs (Fig. 2D). Similarly, the association of β-catenin on the two TCF binding sites (TCFbs 1 and 2) present on this promoter was significantly reduced in KO Mefs (Fig. 2E), suggesting that Copr5/Prmt5 is required for β-catenin recruitment and TCF-mediated transcriptional repression of *Dlk-1*. Of note, we failed to detect a direct protein-protein interaction between Copr5/Prmt5 and β-catenin *in vitro* (data not shown). Interestingly, we found that the recruitment of Brg-1, a chromatin remodeller that can be recruited by β-catenin to TCF target gene promoters and able to interact with Prmt5 (Curtis and Griffin, 2012; de la Serna et al., 2001; Griffin et al., 2011), decreased slightly in KO compared to WT cells (supplementary material Fig. S4). To which extent proteins that are able to antagonise β-catenin/TCF activity might be responsible of this reduced binding of β-catenin at the *Dlk-1* promoter in KO cells still remains. We next assessed whether a shRNA-mediated depletion of *Dlk-1* could restore the capacity of these cells to differentiate. Because they differentiated poorly once infected with shRNAs, we used Copr5-depleted F442A cells. Although a reduction of *Dlk-1* level was obtained in these cells, this reduction was still unable to fully restore a capacity of differentiation (Fig. 2F,G). Hence, this indicates that the impact of *Copr5* on the early step of adipocyte differentiation does not rely exclusively on *Dlk-1*.

Altogether our data identify Copr5 as a negative regulator of *Dlk-1* gene expression in Mefs and suggest that Copr5/Prmt5 recruitment on the *Dlk-1* promoter is a prerequisite for the β-catenin binding and formation of a functional TCF-associated repressor complex on this promoter.

### Upregulation of *Dlk-1* expression in progenitor cell containing VSF of the WAT and modification of the adipocyte cellularity in *Copr5* KO mice

**Fig. 3.**
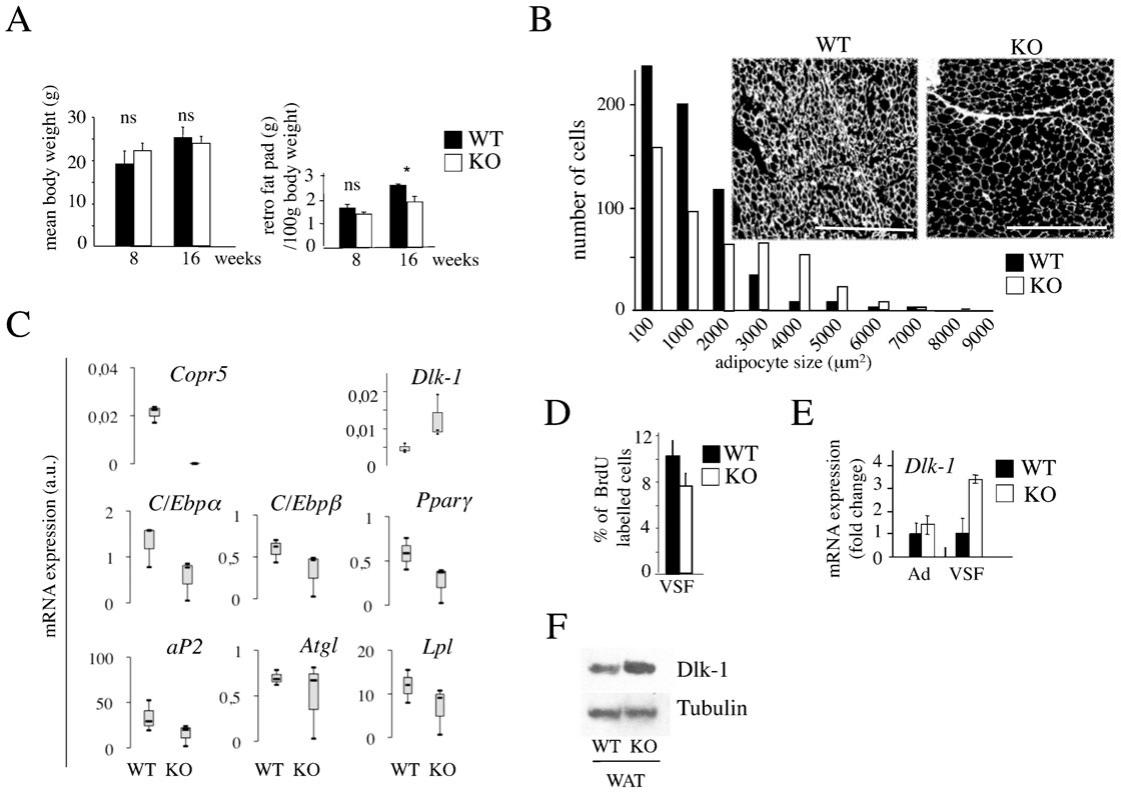
Modification of adipocyte cellularity in adipose tissue of *Copr5* KO mice. (A) Histograms showing the total (left panel) and retroperitoneal fat (right panel) weights in WT and KO mice at 8 and 16 weeks of age (n = 5). *p<0.1 Student's t test; ns, not significant. (B) Histogram showing the size distribution of adipocytes from WT and KO mice. Measurements were performed on equivalent frames using the ImageJ software. Data are representative of three independent mice. A digitalised image of WAT sections from WT and KO mice stained with haematoxylin/eosin is presented. Scale bar: 125 µm. (C) RNA from adipose tissue of WT and KO mice was used to assess by RT-qPCR the expression profile of different adipogenic markers, as indicated. Result expressed in arbitrary units (a.u.) were normalised to *S26* RNA and is the mean±s.d. of three independent mice. (D) Histogram from FACS analysis showing the percentage of BrdU incorporation in SVF cells one week after peritoneal injection of the marker in WT and KO mice (n = 4). (E) RNA from VSF and mature adipocytes (Ad) isolated from 8 week/old KO and WT mice was extracted and assessed as in (C) to analyse *Dlk-1* expression. (F) Western blot of WAT protein extracts from WT and KO mice using anti-Dlk-1 membrane isoform and anti-Tubulin antibodies is shown. In C, the box-plot representation shows the median value of mRNA expression (bold line), the lower and upper limits of each box representing the first and third quartiles, respectively. Whiskers represent the limits of extreme measurements (n = 3). For A,D,E, values are expressed as a mean±s.e.m. (n = 5, n = 4 and n = 4, respectively).

Knockout and WT male mice were indistinguishable with a similar mean body weight (Fig. 3A, left panel). Histological analysis did not reveal significant morphological changes (C.P., E.F., data not shown), excepted in retroperitoneal adipose tissue. A more in depth analysis revealed that its mass was moderately, but reproducibly decreased in KO compared to controls at 16 weeks of age (Fig. 3A, right panel) and that it contained fewer adipocytes but of larger size than WT tissue (Fig. 3B). Of note, glucose and insulin tolerance tests were similar in both types of mice, ruling out major alteration of the glucose and insulin-dependent metabolic axis (data not shown). In agreement with our findings in ES cells and Mefs, and consistent with a reduction in the adipocyte number, a phenotype also encountered in transgenic mice for *Dlk-1* (Lee et al., 2003), the mRNA levels of *aP2*, *Lpl*, *C/Ebpα*, *C/Ebp* and *Pparγ* were downregulated, whereas that of *Dlk-1* was upregulated in KO WAT, supporting a role of *Copr5* in controlling adipogenesis *in vivo* (Fig. 3C). Hence, we hypothesised that the large adipocytes detected in KO mice could reflect an adaptive response to a reduced proliferation/differentiation of KO preadipocytes. Consistently, the proliferation index of the VSF, a main source of progenitor cells, was lower in KO compared to WT mice (Fig. 3D) and associated with a strong upregulation of *Dlk-1* expression, whereas no difference was noted in mature adipocytes (Ad), as expected (Fig. 3E). In addition, we found that the Dlk-1 membrane-bound isoform (Dlk-1) which exerts a negative effect on preadipocyte proliferation (Mortensen et al., 2012), was increased in WAT (Fig. 3F).

**Fig. 4.**
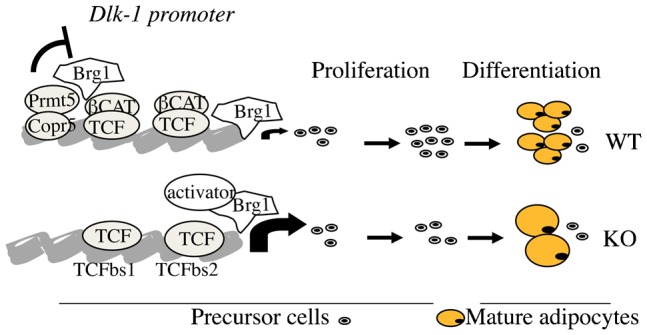
Schematic diagram recapitulating the transcriptional regulation on the Dlk-1 promoter. See Results and Discussion for explanations.

Altogether, our data suggest a model depicted in Fig. 4 in which modification of the adipocyte cellularity observed in KO WAT is a consequence of *Dlk-1* upregulation, leading consequently to a low pool of precursor cells that is able to differentiate into adipocytes. This impaired adipocyte differentiation resulted, at least in part, from a reduced recruitment of Prmt5, Brg1 and β-catenin to the *Dlk-1* promoter in *Copr5* KO mice.

Further studies are now required to understand whether Prmt5/Copr5 complex participates in the transcriptional regulation of other β-catenin-regulated genes that are deregulated in KO Mefs. It will be also interesting to explore whether this complex controls *Dlk-1* expression in the other few adult tissue/glands/neurons that maintain an expression of *Dlk-1*, and whether a deregulation of its expression generated subtle and yet unidentified phenotypes in these organs.

## MATERIALS AND METHODS

### Cell culture conditions

Adipogenic differentiation was induced in post-confluent cells upon addition of a differentiation cocktail (50 nM insulin, 0.5 mM IBMX, 1 mM dexamethasone and 10^−6^ M rosiglitazone) to the medium. ES cells and EBs were cultivated as described previously (Dani et al., 1997).

### Mice and animal care

Animal experiments were approved by the Ethics Committee of the Languedoc-Roussillon Region (France).

### Vascular stromal fraction (VSF) isolation

Adipose tissue was dissected, washed in PBS with a 2% penicillin/streptomycin/gentamycin mixture, minced, and incubated in DMEM supplemented with 10 mg/ml BSA, 0.35% type II collagenase (SIGMA) at 37°C with shaking for 30 min. Cell suspensions were obtained after filtration through 100 µm cell strainers, centrifuged. The remaining pellet was resuspended, filtered through 40 µm cell strainers and centrifuged to recover the VSF.

### Flow cytometry

*In vivo* labelling was performed by intraperitoneal injection of either BrdU at a concentration of 50 µg BrdU/g body weight or PBS included as negative controls in 7-week-old animals that were sacrificed seven days later for VSF isolation. VSF was processed using the BrdU FITC kit, as recommended by BD Pharmingen.

### Determination of the adipocyte size

Sections of paraffin-embedded adipose tissue were stained with haematoxylin/eosin. Quantification was performed from images within a 500×500 µm measurement frame using ImageJ. Three independent measurements were performed in both WT and KO mice (n = 3).

### RNA isolation, cDNA synthesis and RT-qPCR amplification

RNA isolation and RT-qPCR were performed as described (Paul et al., 2012). The sequence of the oligonucleotides is listed in supplementary material Table S2.

### Chromatin immunoprecipitation

Anti-Prmt5 and -β-catenin (S33/37/41) antibodies (Euromedex and Cell Signaling, respectively) were used for ChIP, as described (Paul et al., 2012). Sequence of the oligonucleotides is listed in supplementary material Table S2.

### Western blot

Anti-Prmt5 (Millipore), -β-catenin (Cell Signaling), Histone H3 (Millipore), -Dlk-1 (Abcam) and -Tubulin antibodies were used.

### Microarray analysis

Microarray analysis was performed using total RNA isolated from either Copr5 KO or WT Mefs from male embryos (n = 3), hybridised onto a GeneChip® Mouse Gene 2.0 ST Array and analysed for differentially expressed genes (KFB, Germany) that were considered significant when the *Z* ratio and the adjusted Z*p* value was >2 and 0.05, respectively.

## Supplementary Material

Supplementary Material
