# Supplementary material for: The Wnt-target gene Dlk-1 is regulated by the Prmt5-associated factor Copr5 during adipogenic conversion
Source: Biol Open. 2015 Feb 13;4(3):312–6. doi: 10.1242/bio.201411247 (PMC4359737; doi:10.1242/bio.201411247)
Supplement: Supplementary Material [file supp_4_3_312__index.html]

The Wnt-target gene Dlk-1 is regulated by the Prmt5-associated factor Copr5 during adipogenic conversion — Supplementary Material 

# The Wnt-target gene *Dlk-1* is regulated by the Prmt5-associated factor Copr5 during adipogenic conversion

## bio.201411247 Supplementary Material

**Files in this Data Supplement:**

- Supplementary Material - Conception Paul et al. doi: 10.1242/bio.201411247
- Table S1 - List of differentially expressed genes between WT and Copr5 KO MEFs.
