# Supplementary material for: The Wnt-target gene Dlk-1 is regulated by the Prmt5-associated factor Copr5 during adipogenic conversion
Source: Biol Open. 2015 Feb 13;4(3):312–6. doi: 10.1242/bio.201411247 (PMC4359737; doi:10.1242/bio.201411247)
Supplement: Supplementary Material [file supp_bio.201411247_bio.201411247-s1.pdf]

Biology Open (2015) 000, 1–5 doi:10.1242/bio.201411247

Supplementary Material

Conception Paul et al. doi: 10.1242/bio.201411247

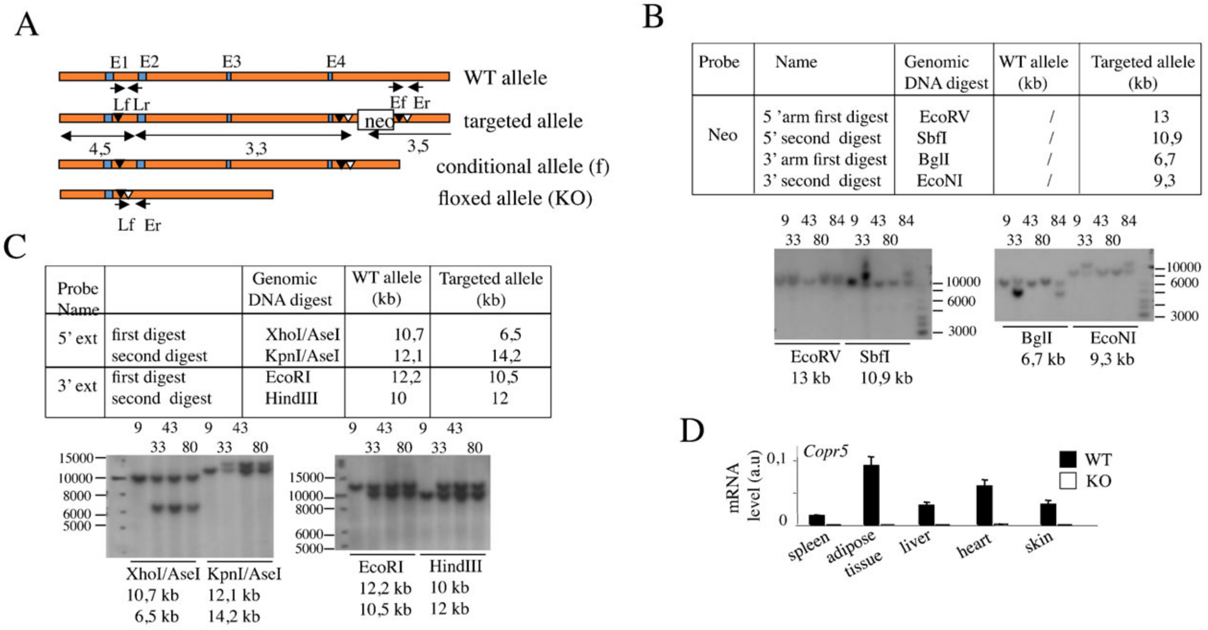

B

Probe	Name	Genomic DNA digest	WT allele (kb)	Targeted allele (kb)
Neo	5 'arm first digest	EcoRV	/	13
	5' second digest	SbfI	/	10,9
	3' arm first digest	BglI	/	6,7
	3' second digest	EcoNI	/	9,3

C

Probe Name		Genomic DNA digest	WT allele (kb)	Targeted allele (kb)
5' ext	first digest	XhoI/AseI	10,7	6,5
	second digest	KpnI/AseI	12,1	14,2
3' ext	first digest	EcoRI	12,2	10,5
	second digest	HindIII	10	12

Fig. S1. Targeting strategy and validation of the *Copr5*^{f} allele. (A) Diagram of the mouse *Copr5* gene. *Copr5* gene was targeted by homologous recombination and a Cre-loxP strategy. Embryonic stem cells were generated in which exons E2 to E4 of *Copr5* were flanked by two loxP sites. To obtain *Copr5 floxed/floxed* (*Copr5* KO) mice, loxed exons were deleted by breeding *wild type/flox* (*WT/f*) mice carrying the recombined allele with CMV-Cre mice, which led to the generation of a heterozygous null allele. Then, we performed *Copr5 WT/floxed* crosses and obtained *Copr5* KO mice. Of note, deletion of E2 to E4 is expected to produce a shorter mRNA that, if stable, would give rise to a Copr5 protein truncated at amino acid 23. Consequently, the Prmt5 binding domain localised previously between amino acids 160 and 184 (full length Copr5 protein consists of 184 amino acids) is absent in KO mice, thereby eliminating Copr5-mediated Prmt5 functions, as published in Lacroix et al., 2008. The positions of the LoxP (black triangles) and FRT (white triangles) sites surrounding exons 2 to 4 and the *neo* marker are shown, respectively. Black arrows indicate the positions of the primers used for genotyping by PCR. (B) Southern Blot Neo: 5′ and 3′ arm validation. Four different digests are used to validate correct homologous recombination event. Two digests validate the 5′ insertion, two other digests validate the 3′ insertion, as indicated. (C) Southern Blot with external 5′ and 3′ probes. Two different digests were used to validate 5′ and 3′ arm, as indicated. From (B) and (C), correctly targeted clone 43 was injected into C57BL6/J blastocysts. (D) Beside genotyping identification by PCR using Lf, Er, Ef and Er primers (not shown), analysis of *Copr5* mRNA level in different tissues from WT and *Copr5* KO mice confirmed that mice were invalidated for *Copr5*.

Biology Open (2015) 000, 1–5 doi:10.1242/bio.201411247

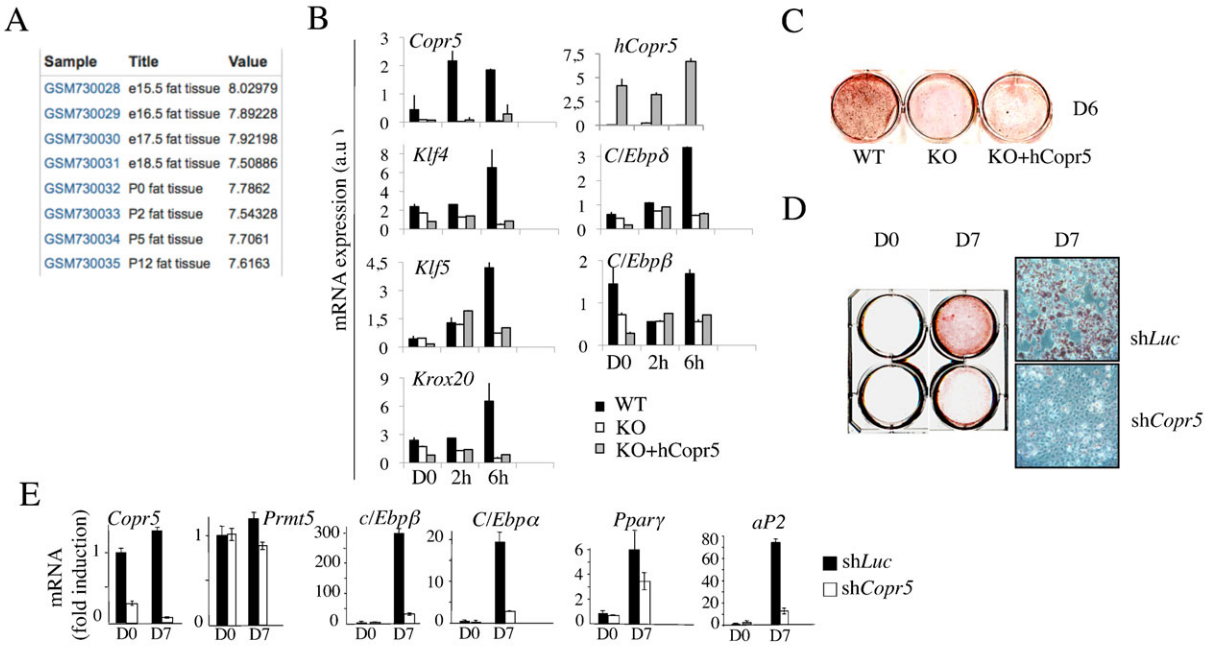

Fig. S2. Impaired adipogenic conversion in Copr5-depleted cells. (A) Expression level of *Copr5* mRNA during development in fat tissue was extracted from previously published Illumina data (Birsoy et al., 2011). (B) Expression level of very early adipogenic markers was assessed in WT, *Copr5* KO Mefs and *Copr5* KO Mefs overexpressing a human form of *Copr5* (*hCopr*5) and monitored by RT-qPCR at early time point of differentiation. Values were normalised to S26 RNA expression. Values expressed in arbitrary units (a.u.) are the mean±s.e.m. of three independent experiments. (C) As a cue of perturbation of adipocyte conversion, plates seeded in parallel with the three different populations of Mefs in (B) were visualised after O Red Oil staining at day 6 (D6) of differentiation. (D) Phase contrast micrographs of post-confluent (D0) and differentiated (D7) F442A cells transduced with *LUC* (sh*Luc*) or *Copr5 (shCopr5)* shRNAs. Cells were induced to differentiate into adipocytes by addition of 50 nM Insulin and 10^{-6} M rosiglitazone at D0. Differentiation was visualised after O Red Oil staining. (E) Expression of mRNA in F442A cells transduced as in (D) was monitored by RT-qPCR and is shown at D0 and D7 of differentiation. Values normalised to S26 RNA expression and expressed as fold change compared to control are the mean±s.e.m. of three independent experiments.

Biology Open

Biology Open (2015) 000, 1–5 doi:10.1242/bio.201411247

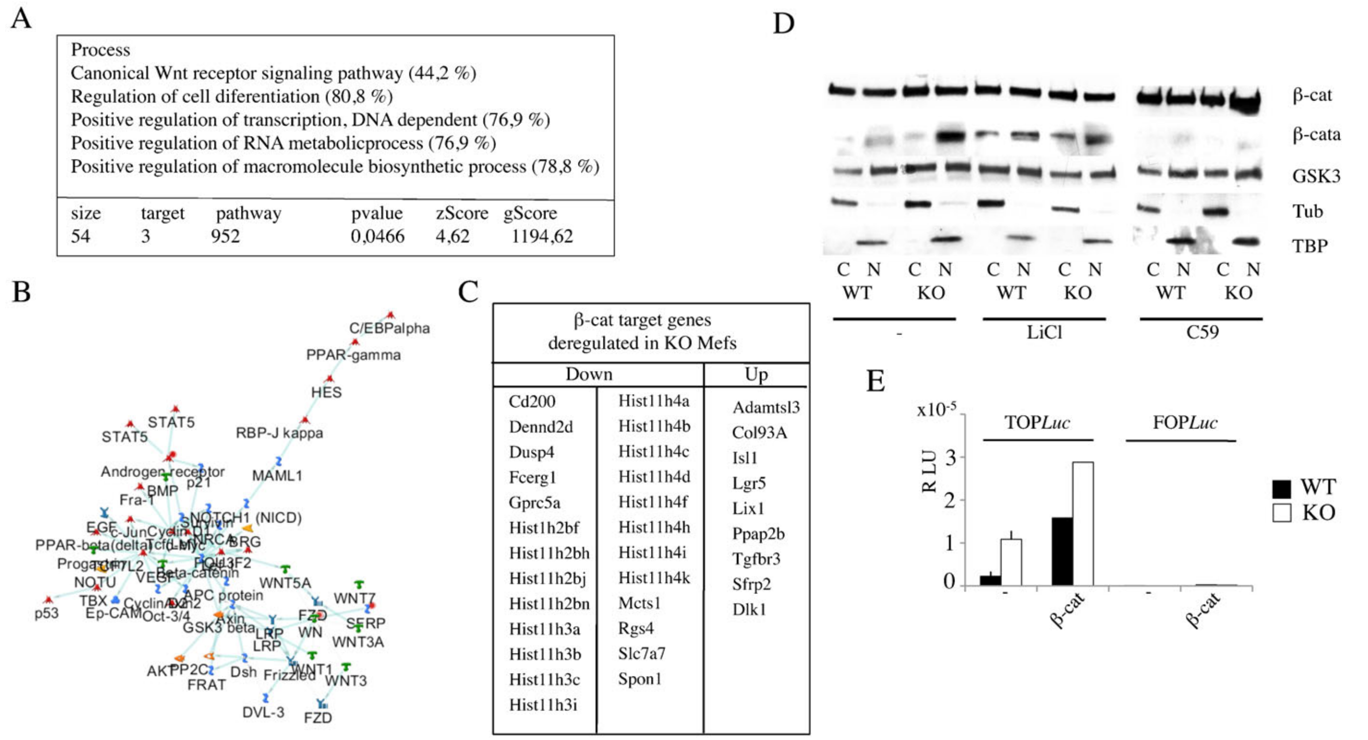

Fig. S3. β-catenin signaling pathway is activated in KO Mefs. (A) Processes involved in KO Mefs are shown. (B) The most relevant network identified with Metacore analysis is shown. The list of significantly deregulated genes obtained from comparative transcriptome analysis between WT and KO Mefs was used for generation of biological networks using Analyze Network algorithm with default settings. This is a variant of the shortest path algorithm with main parameters of 1. relative enrichment with the data, and 2. relative saturation of network with canonical pathways. This network is built on the fly and unique for these data. The network is prioritised based on the number of fragments of canonical pathway of the network. Thick cyan lines indicate the fragments of canonical pathways. Up-regulated genes are marked with red circles; downregulated with blue circles. (C) Sublist of deregulated genes in KO Mefs that are Wnt/β-catenin target genes. (D) Western blot analysis using indicated antibodies was performed on cytoplasmic and nuclear extracts of WT and KO Mefs. Cells were either untreated or treated for 24 h with lithium (LiCl 10 mM) which stabilises β-catenin, or treated with C59 (10 μg/ml) (Abcam), an inhibitor of the Wnt pathway that prevents Wnt secretion. (E) WT and KO Mefs were transfected with CMV-βgal and Luciferase reporters driven by either four synthetic wild type (TOP*Luc*) or mutated (FOP*Luc*) TCF binding sites in the presence or the absence of a plasmid encoding for a stabilised form of β-catenin (β-catS33Y). Results normalised to β-galactosidase activity are expressed as relative luciferase activity (RLU) and show that high level of activated β-catenin was present in KO cells.

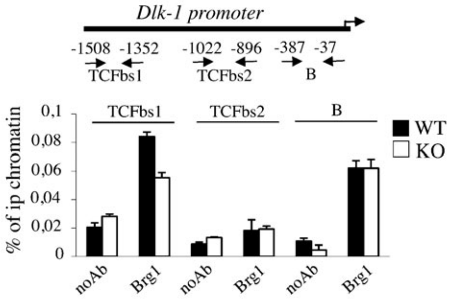

Fig. S4. Brg1 recruitment to the *Dlk-1* promoter is slightly impaired in KO Mefs. Brg1 recruitment to TCFbs1, TCFbs2 and B regions of the *Dlk-1* promoter in WT and KO Mefs was analysed by ChIP. Values are expressed as the percentage of immunoprecipitated (ip) chromatin relative to input and are the mean±s.e.m. of triplicates. No antibody (noAb) was used as negative control. The recruitment of Brg1 at TCFbs1 but not TCFbs2 in WT cells decreased slightly in KO cells, whereas that observed at region B was Copr5/Prmt5-independent and might be allotted for activation.

Biology Open

Biology Open (2015) 000, 1–5 doi:10.1242/bio.201411247

Table S1: See supplementary webpage

Table S2. Oligonucleotides used for RT-qPCR and and Chlp PCR

Oligonucleotides used for RT-qPCR

Pparγ: 5'fCGGGGTAGTCACCATTAACA, 5'rCTTTTTGCCATTGCCACAGA;

C/Ebpα: 5'fGCTGTTATGGGTGAAACTCT, 5'rTGGCATCTCTGTGTCAACCA;

C/Ebpβ: 5'fAGTCGGTGGACAAGAACAGC, 5'rACTCCAGCACCTTCTGTTGC;

C/EBPδ: 5'fTCTCTGACAGGTGGGGAGT, 5'rGCTGCCAGAGGTGGCACT;

aP2: 5'fAACACCGAGATTTCCTTCAA, 5'rAGTCACGCCTTTCATAACACA;

Fas: 5'fTGCTCCCAGCTGCAGGC, 5'rGCCCGGTAGCTCTGGGTGTA;

Atgl: 5'fGAGCCCCGGGGTGGAACAAGAT, 5'rAAAAGGTGGTGGGCAGGAGTAAGG;

Lpl: 5'fACCAAGCTGGTGGGAAATGATGTG, 5'rCCCAGCTGGATCCAAACCAGTAAT;

Gata6: 5'fGAGCTGGTGCTACCAAGAGG, 5'rACGAACGCTTGTGAAATGTG;

Myf5: 5'fACAGCAGCTTTGACAGCATC, 5'rAAGCAATCCAAGCTGGACAC;

Dlk-1: 5'fGAAATAGACGTTCGGGCTTG, 5'r AGGGAGAACCATTGATCACG

Oligonucleotides used for ChIp PCR

Dlk-1 TCFbs1 5f' TGGAGATTAAATTCAAGCTGTCAG, 5'r GCAGCCAAACTTGAGTTTGATC ;

Dlk-1 TCFbs2 5'f CATTTGACGGTGAACATATTGG-3', 5'r GCCCAGACCCCAAATCTGTC ;

Dlk-1 region B (−381/−37) 5'f-GCGCGGGACTCCAGCCCTAAGT,

5'r-GCGGTGCAGGGGCTGCTCCGGG,

Dlk-1 region A (-2263/-2143) *5'f* TGTCTAACCACCCTACCTCAAA,

5'r CTCTGAGAAAAGATGTTGGGATTT

Biology Open
