# Supplementary material for: The Wnt-target gene Dlk-1 is regulated by the Prmt5-associated factor Copr5 during adipogenic conversion
Source: Biol Open. 2015 Feb 13;4(3):312–6. doi: 10.1242/bio.201411247 (PMC4359737; doi:10.1242/bio.201411247)
Supplement: Supplementary Material [file supp_bio.201411247_Table_S1.docx]

| **Table S1. List of differentially expressed genes between WT and Copr5 KO MEFs** | | | |
| --- | --- | --- | --- |
|  |  |  |  |
|  |  |  |  |
| Gene.Accession | Gene.Symbol | ZRatio | ZpValues |
| UP-REGULATED GENES | |  |  |
| NM_008362 | Il1r1 | 2,00E+00 | 5,39E-02 |
| ENSMUST00000019051 | Alox12e | 2,00E+00 | 5,37E-02 |
| ENSMUST00000084838 | Cd47 | 2,00E+00 | 5,36E-02 |
| NM_025524 | Krtap3-3 | 2,01E+00 | 5,30E-02 |
| ENSMUST00000107166 | Odz4 | 2,01E+00 | 5,25E-02 |
| ENSMUST00000119129 | Cdon | 2,02E+00 | 5,14E-02 |
| ENSMUST00000107764 | Hemgn | 2,03E+00 | 5,12E-02 |
| ENSMUST00000056571 | Syne1 | 2,03E+00 | 5,07E-02 |
| ENSMUST00000065858 | Nlgn3 | 2,03E+00 | 5,06E-02 |
| NM_144817 | Camk1g | 2,03E+00 | 5,06E-02 |
| NM_001113736 | Gm13040 | 2,03E+00 | 5,06E-02 |
| AK136967 | Gm10808 | 2,03E+00 | 5,05E-02 |
| ENSMUST00000096433 | Deptor | 2,03E+00 | 5,03E-02 |
| NR_039597 | Mir5135 | 2,04E+00 | 5,02E-02 |
| ENSMUST00000132527 | Col9a3 | 2,04E+00 | 5,00E-02 |
| NR_002847 | Malat1 | 2,04E+00 | 5,00E-02 |
| NR_015582 | C330024D21Rik | 2,04E+00 | 4,99E-02 |
| NM_146810 | Olfr912 | 2,04E+00 | 4,99E-02 |
| NM_024291 | Ky | 2,04E+00 | 4,97E-02 |
| ENSMUST00000101021 | LOC100862180 LOC100862166 Gm7982 Gm7682 LOC624931 Gm6351 | 2,06E+00 | 4,82E-02 |
| NM_008435 | Kcns1 | 2,06E+00 | 4,81E-02 |
| XM_003085040 | Gm2959 | 2,06E+00 | 4,76E-02 |
| NM_001113736 | Gm13040 | 2,06E+00 | 4,75E-02 |
| NM_199301 | Mtg1 | 2,06E+00 | 4,73E-02 |
| NM_198302 | Rbm11 | 2,07E+00 | 4,70E-02 |
| NM_201255 | Krt9 | 2,07E+00 | 4,68E-02 |
| NM_013786 | Hsd17b6 | 2,08E+00 | 4,59E-02 |
| AK086339 | Gm20382 | 2,08E+00 | 4,56E-02 |
| NR_029761 | Mir328 | 2,08E+00 | 4,55E-02 |
| XR_108184 | Gm6909 | 2,08E+00 | 4,54E-02 |
| NM_001011859 | Olfr965 | 2,09E+00 | 4,49E-02 |
| NM_146632 | Olfr116 | 2,09E+00 | 4,49E-02 |
| NM_009197 | Slc16a2 | 2,09E+00 | 4,45E-02 |
| NM_146640 | Olfr1153 | 2,09E+00 | 4,45E-02 |
| ENSMUST00000172036 | Gm13057 Gm13040 Gm13043 | 2,10E+00 | 4,43E-02 |
| ENSMUST00000042221 | Slc26a7 | 2,10E+00 | 4,37E-02 |
| NM_177743 | Fam198a | 2,11E+00 | 4,34E-02 |
| NR_028300 | 5330426P16Rik | 2,11E+00 | 4,31E-02 |
| NM_021600 | Chrnd | 2,11E+00 | 4,30E-02 |
| NM_009075 | Rpia | 2,11E+00 | 4,29E-02 |
| NM_030699 | Ntng1 | 2,11E+00 | 4,26E-02 |
| BC119078 | E230019M04Rik | 2,12E+00 | 4,25E-02 |
| NM_080433 | Fezf2 | 2,12E+00 | 4,25E-02 |
| ENSMUST00000056571 | Syne1 | 2,12E+00 | 4,24E-02 |
| NM_145463 | Shisa2 | 2,12E+00 | 4,22E-02 |
| ENSMUST00000059667 | Hpcal4 | 2,12E+00 | 4,19E-02 |
| NM_007496 | Zfhx3 | 2,13E+00 | 4,14E-02 |
| NM_030718 | Abo | 2,13E+00 | 4,13E-02 |
| NM_008730 | Nptx1 | 2,13E+00 | 4,12E-02 |
| NM_199473 | Col8a2 | 2,13E+00 | 4,10E-02 |
| NR_046144 | Rn5s20 | 2,13E+00 | 4,09E-02 |
| NM_080555 | Ppap2b | 2,13E+00 | 4,09E-02 |
| ENSMUST00000072789 | Gm10063 | 2,14E+00 | 4,06E-02 |
| NM_024474 | Emid2 | 2,14E+00 | 4,01E-02 |
| ENSMUST00000020448 | Irak3 | 2,14E+00 | 4,00E-02 |
| ENSMUST00000099498 | Ccdc80 | 2,15E+00 | 3,96E-02 |
| NM_146366 | Olfr1093 | 2,15E+00 | 3,94E-02 |
| NM_172851 | Cntnap5b | 2,15E+00 | 3,93E-02 |
| NM_133213 | Xpnpep2 | 2,15E+00 | 3,92E-02 |
| ENSMUST00000112641 | Steap3 | 2,16E+00 | 3,91E-02 |
| ENSMUST00000101021 | LOC100862180 LOC100862166 Gm7982 Gm7682 LOC624931 Gm6351 | 2,16E+00 | 3,86E-02 |
| NR_027836 | D730001G18Rik | 2,17E+00 | 3,82E-02 |
| NM_001136087 | Slc6a18 | 2,17E+00 | 3,76E-02 |
| NR_045729 | Nron | 2,19E+00 | 3,63E-02 |
| XM_003085238 | Gm19956 | 2,19E+00 | 3,61E-02 |
| BC067074 | BC067074 | 2,19E+00 | 3,60E-02 |
| NM_001001332 | BC117090 | 2,20E+00 | 3,55E-02 |
| NM_001252292 | Mest | 2,20E+00 | 3,52E-02 |
| NM_028882 | Sema3d | 2,20E+00 | 3,51E-02 |
| NR_024202 | Rny3 | 2,21E+00 | 3,51E-02 |
| NM_001085412 | Gm13078 | 2,21E+00 | 3,49E-02 |
| NR_039541 | Mir3096b | 2,21E+00 | 3,48E-02 |
| AK019492 | 4632411P08Rik | 2,21E+00 | 3,48E-02 |
| NR_033539 | Gm12298 | 2,22E+00 | 3,42E-02 |
| NM_029131 | 4930503E14Rik | 2,22E+00 | 3,41E-02 |
| NR_030460 | Mir688 | 2,22E+00 | 3,36E-02 |
| NM_001199556 | AW551984 | 2,23E+00 | 3,35E-02 |
| ENSMUST00000070375 | Penk | 2,23E+00 | 3,32E-02 |
| NM_001034115 | Shank1 | 2,23E+00 | 3,30E-02 |
| NM_146498 | Olfr490 | 2,25E+00 | 3,19E-02 |
| NM_025681 | Lix1 | 2,25E+00 | 3,16E-02 |
| BC151018 | A430107O13Rik | 2,25E+00 | 3,15E-02 |
| NM_009154 | Sema5a | 2,26E+00 | 3,14E-02 |
| NM_001252070 | Dnahc7a | 2,26E+00 | 3,10E-02 |
| NM_009675 | Aoc3 | 2,27E+00 | 3,06E-02 |
| ENSMUST00000001181 | Col6a2 | 2,27E+00 | 3,05E-02 |
| NM_028402 | Zfp493 | 2,27E+00 | 3,05E-02 |
| NM_020052 | Scube2 | 2,27E+00 | 3,04E-02 |
| NR_035476 | Mir1951 | 2,27E+00 | 3,04E-02 |
| NM_025809 | Clec14a | 2,27E+00 | 3,03E-02 |
| NM_001040699 | Mtmr7 | 2,27E+00 | 3,02E-02 |
| NM_018867 | Cpxm2 | 2,28E+00 | 2,96E-02 |
| NM_007470 | Apod | 2,28E+00 | 2,94E-02 |
| ENSMUST00000169130 | Vmn2r62 | 2,28E+00 | 2,93E-02 |
| NM_134022 | 6330403K07Rik | 2,29E+00 | 2,88E-02 |
| NR_035499 | Mir1971 | 2,29E+00 | 2,87E-02 |
| NM_010290 | Gjd2 | 2,30E+00 | 2,86E-02 |
| ENSMUST00000169949 | Gm9140 Gm5632 | 2,30E+00 | 2,81E-02 |
| NM_010097 | Sparcl1 | 2,31E+00 | 2,78E-02 |
| ENSMUST00000130418 | Aldh1l1 | 2,31E+00 | 2,76E-02 |
| NM_027928 | Chst13 | 2,31E+00 | 2,76E-02 |
| ENSMUST00000056571 | Syne1 | 2,31E+00 | 2,74E-02 |
| NM_001162950 | Hif3a | 2,32E+00 | 2,71E-02 |
| NM_001081072 | Slc27a6 | 2,32E+00 | 2,69E-02 |
| NM_207624 | Ace | 2,32E+00 | 2,69E-02 |
| NM_146913 | Olfr1348 | 2,32E+00 | 2,69E-02 |
| NM_001011784 | Olfr1039 | 2,34E+00 | 2,59E-02 |
| ENSMUST00000095049 | Mup15 | 2,34E+00 | 2,59E-02 |
|  | Mup2 |  |  |
| NM_021381 | Prokr1 | 2,34E+00 | 2,59E-02 |
| NM_009131 | Clec11a | 2,34E+00 | 2,58E-02 |
| NM_020595 | Otor | 2,34E+00 | 2,56E-02 |
| NM_030708 | Zfhx4 | 2,35E+00 | 2,52E-02 |
| NM_175549 | Robo2 | 2,35E+00 | 2,50E-02 |
| ENSMUST00000107392 | Anpep | 2,36E+00 | 2,48E-02 |
| NM_001166658 | Cdr1 | 2,36E+00 | 2,44E-02 |
| NM_134436 | Vmn1r27 | 2,37E+00 | 2,42E-02 |
| NM_001081064 | Pdzd2 | 2,37E+00 | 2,42E-02 |
| NM_021459 | Isl1 | 2,37E+00 | 2,40E-02 |
| NM_001001488 | Atp8b1 | 2,37E+00 | 2,40E-02 |
| BC147525 | 1700123K08Rik | 2,37E+00 | 2,38E-02 |
| NM_010517 | Igfbp4 | 2,38E+00 | 2,33E-02 |
| NM_173385 | Cilp | 2,39E+00 | 2,31E-02 |
| ENSMUST00000056571 | Syne1 | 2,39E+00 | 2,27E-02 |
| NM_007743 | Col1a2 | 2,40E+00 | 2,25E-02 |
| NM_001085528 | Gm13271 | 2,40E+00 | 2,24E-02 |
| NM_177361 | Ifna12 | 2,40E+00 | 2,23E-02 |
| NM_029536 | Gpr165 | 2,42E+00 | 2,12E-02 |
| NM_028903 | Scara5 | 2,43E+00 | 2,06E-02 |
| ENSMUST00000062202 | Sned1 | 2,44E+00 | 2,01E-02 |
| NM_178929 | Kazald1 | 2,45E+00 | 2,00E-02 |
| NM_026058 | Lass4 | 2,45E+00 | 2,00E-02 |
| NM_010550 | Il11ra2 | 2,45E+00 | 1,99E-02 |
| NR_028428 | 2610005L07Rik | 2,45E+00 | 1,97E-02 |
| NM_007706 | Socs2 | 2,45E+00 | 1,97E-02 |
| ENSMUST00000108047 | Tbx4 | 2,46E+00 | 1,94E-02 |
| NM_029106 | Gm14354 | 2,47E+00 | 1,91E-02 |
| NM_007603 | Capn6 | 2,48E+00 | 1,85E-02 |
| NR_033813 | Dlk1 | 2,48E+00 | 1,82E-02 |
| NM_008489 | Lbp | 2,49E+00 | 1,80E-02 |
| NM_001038996 | Try10 | 2,51E+00 | 1,70E-02 |
| NM_001082543 | Stfa1 | 2,52E+00 | 1,67E-02 |
| NR_045335 | Gm13582 | 2,53E+00 | 1,63E-02 |
| ENSMUST00000002360 | Angptl4 | 2,53E+00 | 1,61E-02 |
| NM_001256005 | Gbp4 | 2,54E+00 | 1,58E-02 |
| NR_035497 | Mir1970 | 2,54E+00 | 1,57E-02 |
| AK173199 | Rnf213 | 2,54E+00 | 1,56E-02 |
| NM_030206 | Cygb | 2,55E+00 | 1,53E-02 |
| BC038501 | A830039N20Rik | 2,56E+00 | 1,52E-02 |
| NM_172815 | Rspo2 | 2,56E+00 | 1,52E-02 |
| NM_009144 | Sfrp2 | 2,56E+00 | 1,52E-02 |
| NM_010681 | Lama4 | 2,56E+00 | 1,50E-02 |
| NM_172874 | Podn | 2,56E+00 | 1,50E-02 |
| NM_147048 | Olfr652 | 2,57E+00 | 1,46E-02 |
| NM_013584 | Lifr | 2,57E+00 | 1,45E-02 |
| XR_105215 | Gm2085 | 2,58E+00 | 1,43E-02 |
| NM_139299 | Il31ra | 2,58E+00 | 1,42E-02 |
| NM_001035239 | Trpm3 | 2,59E+00 | 1,40E-02 |
| NM_001177486 | Gm15293 | 2,59E+00 | 1,40E-02 |
| NM_029499 | Ms4a4c | 2,59E+00 | 1,39E-02 |
| NM_026439 | Ccdc80 | 2,59E+00 | 1,38E-02 |
| NM_172454 | Panx3 | 2,60E+00 | 1,37E-02 |
| NM_025817 | Tril | 2,60E+00 | 1,35E-02 |
| NM_033616 | Csprs | 2,61E+00 | 1,34E-02 |
| NM_001171010 | Slc14a1 | 2,61E+00 | 1,33E-02 |
| NM_001081150 | Lonrf1 | 2,62E+00 | 1,30E-02 |
| NM_178715 | Tmem30b | 2,62E+00 | 1,29E-02 |
| NM_146072 | Grik1 | 2,63E+00 | 1,27E-02 |
| NM_025778 | Bcl2l14 | 2,64E+00 | 1,24E-02 |
| NM_153577 | AI428936 | 2,64E+00 | 1,22E-02 |
| AK003924 | 1110025L11Rik | 2,64E+00 | 1,22E-02 |
| NM_173008 | A430110N23Rik | 2,64E+00 | 1,21E-02 |
| XR_140874 | LOC100861653 | 2,65E+00 | 1,19E-02 |
| NM_011674 | Ugt8a | 2,65E+00 | 1,19E-02 |
| NM_173016 | Vat1l | 2,66E+00 | 1,16E-02 |
| NR_033570 | Gm7104 | 2,66E+00 | 1,15E-02 |
| ENSMUST00000003100 | Cyp2f2 | 2,68E+00 | 1,11E-02 |
| BC027570 | Gm3086 | 2,68E+00 | 1,09E-02 |
| NM_001162943 | Dchs1 | 2,71E+00 | 1,01E-02 |
| NM_177794 | Tmem26 | 2,72E+00 | 9,98E-03 |
| NM_010181 | Fbn2 | 2,74E+00 | 9,28E-03 |
| NM_172471 | Itih5 | 2,76E+00 | 8,95E-03 |
| ENSMUST00000078229 | Pou3f4 | 2,76E+00 | 8,74E-03 |
| ENSMUST00000022921 | Angpt1 | 2,78E+00 | 8,27E-03 |
| NM_019397 | Egfl6 | 2,78E+00 | 8,26E-03 |
| XM_003688854 | LOC100862211 | 2,78E+00 | 8,26E-03 |
| NR_037281 | Mir3098 | 2,79E+00 | 8,06E-03 |
| NM_177715 | Kctd12 | 2,81E+00 | 7,65E-03 |
| NM_013912 | Apln | 2,82E+00 | 7,56E-03 |
| ENSMUST00000042850 | Svep1 | 2,85E+00 | 6,94E-03 |
| ENSMUST00000027377 | Igfbp5 | 2,86E+00 | 6,66E-03 |
| XM_156070 | Gm4758 | 2,87E+00 | 6,52E-03 |
| NM_054098 | Steap4 | 2,89E+00 | 6,05E-03 |
| ENSMUST00000032501 | Tspan11 | 2,90E+00 | 5,94E-03 |
| ENSMUST00000056571 | Syne1 | 2,93E+00 | 5,47E-03 |
| NM_008987 | Ptx3 | 2,93E+00 | 5,40E-03 |
| ENSMUST00000049681 | Itgbl1 | 2,96E+00 | 5,01E-03 |
| NM_011537 | Tbx5 | 2,96E+00 | 4,92E-03 |
| NM_147120 | Olfr638 | 2,99E+00 | 4,62E-03 |
| NR_029830 | Mir194-2 | 2,99E+00 | 4,62E-03 |
| NM_008524 | Lum | 3,00E+00 | 4,46E-03 |
| NM_001134661 | Gm6406 | 3,01E+00 | 4,34E-03 |
| NM_177839 | Tnn | 3,01E+00 | 4,27E-03 |
| NM_001162977 | Megf6 | 3,02E+00 | 4,23E-03 |
| NR_028524 | Snord38a | 3,04E+00 | 3,98E-03 |
| NM_019588 | Plce1 | 3,04E+00 | 3,89E-03 |
| ENSMUST00000029135 | Acss2 | 3,07E+00 | 3,62E-03 |
| NM_016917 | Slc40a1 | 3,08E+00 | 3,48E-03 |
| NM_008318 | Ibsp | 3,08E+00 | 3,46E-03 |
| NM_183221 | Fat4 | 3,11E+00 | 3,16E-03 |
| NM_173404 | Bmp3 | 3,12E+00 | 3,09E-03 |
| ENSMUST00000051065 | Gprin3 | 3,13E+00 | 2,98E-03 |
| NM_011380 | Six2 | 3,14E+00 | 2,89E-03 |
| ENSMUST00000052837 | Ar | 3,15E+00 | 2,77E-03 |
| NM_001113415 | Ebf3 | 3,16E+00 | 2,74E-03 |
| ENSMUST00000041183 | Meox2 | 3,17E+00 | 2,60E-03 |
| NM_025585 | 1700029F12Rik | 3,23E+00 | 2,16E-03 |
| NR_028571 | Snora17 | 3,24E+00 | 2,11E-03 |
| NM_177872 | Adamts3 | 3,27E+00 | 1,89E-03 |
| ENSMUST00000057784 | Slc7a2 | 3,29E+00 | 1,80E-03 |
| NM_025759 | Speer4d | 3,29E+00 | 1,77E-03 |
| NM_008607 | Mmp13 | 3,29E+00 | 1,77E-03 |
| NR_030532 | Mir743 | 3,29E+00 | 1,75E-03 |
| ENSMUST00000159861 | Pappa2 | 3,32E+00 | 1,59E-03 |
| NM_011516 | Sycp1 | 3,38E+00 | 1,32E-03 |
| NM_001170954 | A4galt | 3,40E+00 | 1,22E-03 |
| NM_001243008 | Col6a3 | 3,41E+00 | 1,21E-03 |
| NM_001162884 | Igsf10 | 3,49E+00 | 9,17E-04 |
| NM_001085477 | Olfr765 | 3,50E+00 | 8,71E-04 |
| ENSMUST00000031224 | Tgfbr3 | 3,54E+00 | 7,51E-04 |
| NR_028512 | Snora73a | 3,55E+00 | 7,42E-04 |
| NM_001014423 | Abi3bp | 3,55E+00 | 7,33E-04 |
| NM_016685 | Comp | 3,56E+00 | 7,04E-04 |
| NR_028513 | Snora73b | 3,60E+00 | 6,10E-04 |
| NM_001190374 | Adamtsl3 | 3,61E+00 | 5,98E-04 |
| NM_010733 | Lrrn3 | 3,63E+00 | 5,54E-04 |
| ENSMUST00000033198 | Crym | 3,71E+00 | 4,14E-04 |
| ENSMUST00000056571 | Syne1 | 3,71E+00 | 4,04E-04 |
| NM_007606 | Car3 | 3,74E+00 | 3,66E-04 |
| NM_198967 | Tmtc1 | 3,80E+00 | 2,97E-04 |
| NR_028571 | Snora17 | 4,07E+00 | 1,00E-04 |
| ENSMUST00000105285 | Epyc | 4,09E+00 | 9,42E-05 |
| ENSMUST00000065496 | Arhgap20 | 4,16E+00 | 6,96E-05 |
| NM_008604 | Mme | 4,31E+00 | 3,63E-05 |
| NM_013724 | Nrk | 4,37E+00 | 2,79E-05 |
| NM_001083316 | Pdgfra | 4,42E+00 | 2,33E-05 |
| NM_010195 | Lgr5 | 4,45E+00 | 1,98E-05 |
| NM_172907 | Olfml1 | 4,91E+00 | 2,28E-06 |
| NR_028576 | Scarna13 | 5,16E+00 | 6,67E-07 |
| NR_029412 | Snora16a | 5,19E+00 | 5,58E-07 |
| NM_021400 | Prg4 | 5,52E+00 | 9,60E-08 |
| NR_028522 | Snord13 | 6,82E+00 | 3,17E-11 |
| NM_011582 | Thbs4 | 7,07E+00 | 5,77E-12 |
|  |  |  |  |
| DOWN-REGULATED GENES | |  |  |
|  |  |  |  |
| NM_019914 | Mllt11 | 2,00E+00 | 5,39E-02 |
| NM_007904 | Ednrb | 2,01E+00 | 5,33E-02 |
| NM_026902 | Mcts1 | 2,01E+00 | 5,31E-02 |
| NR_045402 | E330013P06 | 2,01E+00 | 5,29E-02 |
| NM_031159 | Apobec1 | 2,01E+00 | 5,29E-02 |
| NR_030271 | Mir487b | 2,01E+00 | 5,29E-02 |
| NM_054045 | Hist2h3c2 | 2,01E+00 | 5,28E-02 |
| AK086282 | Gm19845 | 2,01E+00 | 5,28E-02 |
| NR_030421 | Mir758 | 2,01E+00 | 5,27E-02 |
| NM_134152 | Lpxn | 2,02E+00 | 5,19E-02 |
| NM_001195732 | Fam150a | 2,02E+00 | 5,17E-02 |
| XM_003688876 | LOC100862148 | 2,02E+00 | 5,14E-02 |
| NM_207650 | Dtna | 2,03E+00 | 5,12E-02 |
| NR_028540 | Snord12 | 2,03E+00 | 5,12E-02 |
| NM_053110 | Gpnmb | 2,03E+00 | 5,11E-02 |
| NM_001010838 | Taar7d | 2,03E+00 | 5,08E-02 |
| NR_028523 | Snord19 | 2,04E+00 | 4,96E-02 |
| NM_009246 | Serpina1d | 2,04E+00 | 4,93E-02 |
| NR_035411 | Mir669g | 2,05E+00 | 4,85E-02 |
| ENSMUST00000172388 | Rgs1 | 2,05E+00 | 4,85E-02 |
| NR_029788 | Mir28 | 2,05E+00 | 4,84E-02 |
| NR_028521 | Snord11 | 2,06E+00 | 4,83E-02 |
| AK049386 | C430002N11Rik | 2,06E+00 | 4,82E-02 |
| NM_001252639 | Tbc1d7 | 2,06E+00 | 4,81E-02 |
| ENSMUST00000097477 | LOC100862150 AA792892 | 2,06E+00 | 4,77E-02 |
| NM_183103 | Prss46 | 2,08E+00 | 4,61E-02 |
| NM_010482 | Htr1b | 2,08E+00 | 4,57E-02 |
| NR_024078 | Btbd19 | 2,08E+00 | 4,56E-02 |
| NM_009414 | Tph1 | 2,09E+00 | 4,53E-02 |
| NR_029553 | Mir140 | 2,09E+00 | 4,50E-02 |
| NR_029881 | Mir380 | 2,09E+00 | 4,45E-02 |
| NM_022324 | Sdf2l1 | 2,10E+00 | 4,43E-02 |
| NR_029746 | Mir27a | 2,10E+00 | 4,43E-02 |
| ENSMUST00000075737 | Gm5329 | 2,10E+00 | 4,40E-02 |
| NM_146402 | Olfr1303 | 2,10E+00 | 4,40E-02 |
| NM_001110320 | Cd72 | 2,11E+00 | 4,32E-02 |
| BC116915 | 1700009N14Rik | 2,11E+00 | 4,31E-02 |
| NR_029432 | 1500015A07Rik | 2,11E+00 | 4,30E-02 |
| ENSMUST00000025025 | Dusp1 | 2,11E+00 | 4,28E-02 |
| NM_001177349 | Pydc4 | 2,11E+00 | 4,28E-02 |
| AK161656 | Taf1d | 2,11E+00 | 4,27E-02 |
| NR_030437 | Mir496 | 2,11E+00 | 4,27E-02 |
| NM_013468 | Ankrd1 | 2,12E+00 | 4,25E-02 |
| BC056964 | Taf1d | 2,12E+00 | 4,25E-02 |
| ENSMUST00000033930 | Dusp4 | 2,12E+00 | 4,24E-02 |
| BC007193 | C130026I21Rik | 2,12E+00 | 4,22E-02 |
| NM_013759 | Sepx1 | 2,12E+00 | 4,22E-02 |
| NM_010344 | Gsr | 2,12E+00 | 4,20E-02 |
| NM_009520 | Wnt2b | 2,13E+00 | 4,16E-02 |
| ENSMUST00000043929 | Ccdc68 | 2,13E+00 | 4,15E-02 |
| NM_009987 | Cx3cr1 | 2,14E+00 | 4,04E-02 |
| NM_001030305 | Pmp2 | 2,14E+00 | 4,02E-02 |
| ENSMUST00000005592 | Siglecg | 2,15E+00 | 3,95E-02 |
| NM_026307 | Cuta | 2,16E+00 | 3,91E-02 |
| NM_145136 | Myocd | 2,16E+00 | 3,90E-02 |
| NM_010738 | Ly6a | 2,16E+00 | 3,88E-02 |
| NR_029883 | Mir382 | 2,16E+00 | 3,85E-02 |
| NM_023043 | Prnd | 2,16E+00 | 3,83E-02 |
| NM_016903 | Esd | 2,17E+00 | 3,83E-02 |
| ENSMUST00000147604 | Aim2 | 2,17E+00 | 3,82E-02 |
| ENSMUST00000042603 | Inhba | 2,17E+00 | 3,79E-02 |
| NM_009917 | Ccr5 | 2,18E+00 | 3,73E-02 |
| NR_003270 | Snhg3 | 2,18E+00 | 3,72E-02 |
| NM_007651 | Cd53 | 2,18E+00 | 3,69E-02 |
| AK039813 | Gm15234 | 2,18E+00 | 3,69E-02 |
| NR_029744 | Mir29a | 2,18E+00 | 3,68E-02 |
| NM_146711 | Olfr43 | 2,19E+00 | 3,66E-02 |
| NR_028091 | Snord72 | 2,19E+00 | 3,62E-02 |
| NM_001034862 | Erich1 | 2,19E+00 | 3,62E-02 |
| NM_031254 | Trem2 | 2,19E+00 | 3,60E-02 |
| NM_001099299 | Ajap1 | 2,20E+00 | 3,57E-02 |
| NR_045420 | LOC545261 | 2,20E+00 | 3,56E-02 |
| NM_029478 | Vmp1 | 2,20E+00 | 3,55E-02 |
| NM_008326 | Irgm1 | 2,20E+00 | 3,54E-02 |
| NR_029806 | Mir221 | 2,21E+00 | 3,48E-02 |
| ENSMUST00000102894 | Gp49a | 2,21E+00 | 3,45E-02 |
| NR_033498 | AI504432 | 2,21E+00 | 3,45E-02 |
| NM_146447 | Olfr1309 | 2,21E+00 | 3,44E-02 |
| NM_013563 | Il2rg | 2,22E+00 | 3,38E-02 |
| NM_008726 | Nppb | 2,22E+00 | 3,38E-02 |
| NM_178189 | Hist1h2ac | 2,23E+00 | 3,30E-02 |
| NM_134250 | Havcr2 | 2,23E+00 | 3,29E-02 |
| NM_020008 | Clec7a | 2,24E+00 | 3,28E-02 |
| NR_030473 | Mir669c | 2,24E+00 | 3,27E-02 |
| ENSMUST00000015622 | Rnf5 | 2,24E+00 | 3,27E-02 |
| NR_035411 | Mir669g | 2,24E+00 | 3,25E-02 |
| ENSMUST00000067298 | Mrps21 | 2,24E+00 | 3,23E-02 |
| XM_003085780 | LOC100504941 | 2,24E+00 | 3,21E-02 |
| NM_001005568 | Olfr1281 | 2,24E+00 | 3,21E-02 |
| BC141198 | Ptges3l | 2,25E+00 | 3,21E-02 |
| NM_001105179 | Vmn2r51 | 2,25E+00 | 3,20E-02 |
| BC032201 | Gm5665 | 2,25E+00 | 3,16E-02 |
| NM_001111058 | Cd33 | 2,25E+00 | 3,15E-02 |
| ENSMUST00000137841 | Sumo3 | 2,25E+00 | 3,14E-02 |
| NM_146308 | Olfr1356 | 2,26E+00 | 3,13E-02 |
| NM_008535 | Lyl1 | 2,26E+00 | 3,12E-02 |
| NM_008278 | Hpgd | 2,26E+00 | 3,10E-02 |
| NR_029884 | Mir383 | 2,26E+00 | 3,10E-02 |
| NM_022879 | Myl7 | 2,26E+00 | 3,09E-02 |
| AK085580 | Gm19551 | 2,26E+00 | 3,08E-02 |
| NM_030691 | Igsf6 | 2,27E+00 | 3,02E-02 |
| NR_028479 | Snora20 | 2,27E+00 | 3,01E-02 |
| NR_037231 | Mir3070b | 2,27E+00 | 3,01E-02 |
| AF241256 | Snord116 | 2,28E+00 | 3,00E-02 |
| NR_035452 | Mir1931 | 2,28E+00 | 2,98E-02 |
| NM_026335 | Lce1h | 2,28E+00 | 2,98E-02 |
| NM_146539 | Olfr373 | 2,29E+00 | 2,91E-02 |
| NM_001033435 | Gm885 | 2,29E+00 | 2,90E-02 |
| NR_000003 | Snord35a | 2,31E+00 | 2,78E-02 |
| NR_028560 | Scarna17 | 2,31E+00 | 2,77E-02 |
| ENSMUST00000162731 | Zfp579 | 2,32E+00 | 2,73E-02 |
| NR_029741 | Mir24-2 | 2,32E+00 | 2,72E-02 |
| AK138383 | Gm6712 | 2,32E+00 | 2,72E-02 |
| NR_030253 | Mir485 | 2,33E+00 | 2,67E-02 |
| NM_001024147 | Gm5868 | 2,33E+00 | 2,63E-02 |
| NM_010449 | Hoxa1 | 2,33E+00 | 2,61E-02 |
| NR_029762 | Mir329 | 2,34E+00 | 2,58E-02 |
| NR_029531 | Mir27b | 2,34E+00 | 2,58E-02 |
| NR_002895 | Snord116 | 2,34E+00 | 2,55E-02 |
| NM_023135 | Sult1e1 | 2,34E+00 | 2,55E-02 |
| NM_011405 | Slc7a7 | 2,35E+00 | 2,54E-02 |
| NM_181390 | Mustn1 | 2,36E+00 | 2,49E-02 |
| ENSMUST00000071134 | Tubb3 | 2,36E+00 | 2,46E-02 |
| NM_178196 | Hist1h2bg | 2,36E+00 | 2,44E-02 |
| ENSMUST00000101212 | Nup62cl | 2,37E+00 | 2,43E-02 |
| NR_034047 | Snora19 | 2,38E+00 | 2,36E-02 |
| NR_037244 | Mir3083 | 2,38E+00 | 2,35E-02 |
| NM_177769 | Elmod1 | 2,38E+00 | 2,35E-02 |
| NM_008071 | Gabrb3 | 2,39E+00 | 2,31E-02 |
| NM_013489 | Cd84 | 2,39E+00 | 2,30E-02 |
| NM_008329 | Ifi204 | 2,39E+00 | 2,29E-02 |
| NR_030552 | Mir297a-4 | 2,40E+00 | 2,24E-02 |
| NM_001101533 | Vmn1r20 | 2,40E+00 | 2,23E-02 |
| NM_145584 | Spon1 | 2,40E+00 | 2,22E-02 |
| NM_027571 | P2ry12 | 2,41E+00 | 2,20E-02 |
| NR_029878 | Mir377 | 2,41E+00 | 2,20E-02 |
| NR_040556 | Gm6634 | 2,42E+00 | 2,14E-02 |
| NM_001205355 | Fhdc1 | 2,42E+00 | 2,13E-02 |
| NM_013797 | Slco1a1 | 2,42E+00 | 2,11E-02 |
| NM_001113460 | Tec | 2,42E+00 | 2,11E-02 |
| NR_028541 | Snord65 | 2,43E+00 | 2,07E-02 |
| NM_024223 | Crip2 | 2,44E+00 | 2,05E-02 |
| ENSMUST00000139730 | Top2a | 2,44E+00 | 2,01E-02 |
| NR_029533 | Mir30a | 2,46E+00 | 1,95E-02 |
| NM_147155 | Tagap1 | 2,46E+00 | 1,92E-02 |
| NM_013609 | Ngf | 2,47E+00 | 1,89E-02 |
| NR_051982 | Dennd2d | 2,47E+00 | 1,87E-02 |
| NM_010185 | Fcer1g | 2,48E+00 | 1,86E-02 |
| NM_146015 | Efemp1 | 2,48E+00 | 1,84E-02 |
| NR_030610 | Mir544 | 2,49E+00 | 1,81E-02 |
| NR_029757 | Mir323 | 2,49E+00 | 1,80E-02 |
| NR_039566 | Mir3473c | 2,49E+00 | 1,78E-02 |
| NR_028564 | Snord95 | 2,50E+00 | 1,75E-02 |
| NM_010935 | Npy6r | 2,50E+00 | 1,74E-02 |
| ENSMUST00000139730 | Top2a | 2,50E+00 | 1,74E-02 |
| NM_030110 | Efha2 | 2,52E+00 | 1,69E-02 |
| NM_001122596 | A630033H20Rik | 2,52E+00 | 1,69E-02 |
| NM_023476 | Tinagl1 | 2,52E+00 | 1,68E-02 |
| ENSMUST00000142269 | Gnb2l1 | 2,53E+00 | 1,63E-02 |
| ENSMUST00000049389 | Zdhhc2 | 2,54E+00 | 1,60E-02 |
| NM_010478 | Hspa1b | 2,54E+00 | 1,57E-02 |
| NM_184109 | Rtl1 | 2,54E+00 | 1,57E-02 |
| NR_029557 | Mir145 | 2,55E+00 | 1,53E-02 |
| NM_011408 | Slfn2 | 2,56E+00 | 1,50E-02 |
| NM_022032 | Perp | 2,56E+00 | 1,50E-02 |
| AK035387 | Gm20559 | 2,57E+00 | 1,47E-02 |
| ENSMUST00000102493 | Coro6 | 2,58E+00 | 1,42E-02 |
| NM_009062 | Rgs4 | 2,58E+00 | 1,42E-02 |
| NM_010104 | Edn1 | 2,59E+00 | 1,39E-02 |
| NM_146425 | Olfr470 | 2,59E+00 | 1,39E-02 |
| NR_035472 | Mir1949 | 2,59E+00 | 1,38E-02 |
| NR_030711 | 2210403K04Rik | 2,61E+00 | 1,33E-02 |
| NM_016861 | Pdlim1 | 2,61E+00 | 1,33E-02 |
| NR_029572 | Mir186 | 2,61E+00 | 1,32E-02 |
| NR_030578 | Mir654 | 2,62E+00 | 1,30E-02 |
| ENSMUST00000006392 | Serpinb9b | 2,62E+00 | 1,30E-02 |
| NR_030471 | Mir669a-3 | 2,62E+00 | 1,29E-02 |
| NR_028128 | Snord68 | 2,62E+00 | 1,28E-02 |
| ENSMUST00000024706 | Pla2g7 | 2,63E+00 | 1,25E-02 |
| NM_021367 | Tslp | 2,63E+00 | 1,24E-02 |
| NM_178610 | Krr1 | 2,65E+00 | 1,19E-02 |
| ENSMUST00000035158 | Trf | 2,66E+00 | 1,15E-02 |
| NR_029457 | G530011O06Rik | 2,67E+00 | 1,13E-02 |
| NM_010819 | Clec4d | 2,67E+00 | 1,12E-02 |
| ENSMUST00000151871 | LOC100505283 Gm15682 Gm10045 Gm11703 LOC634339 Gm13653 Gm5445 | 2,69E+00 | 1,06E-02 |
|  | Rpl21 |  |  |
| BC066104 | 9930111J21Rik2 | 2,70E+00 | 1,04E-02 |
| NR_029795 | Mir181a-1 | 2,70E+00 | 1,04E-02 |
| ENSMUST00000025319 | Rpp21 | 2,72E+00 | 9,94E-03 |
| NM_010745 | Ly86 | 2,72E+00 | 9,91E-03 |
| NR_029820 | Mir181b-1 | 2,72E+00 | 9,91E-03 |
| NR_030426 | Mir667 | 2,73E+00 | 9,54E-03 |
| NR_029575 | Mir24-1 | 2,74E+00 | 9,46E-03 |
| NM_008372 | Il7r | 2,74E+00 | 9,36E-03 |
| NR_028560 | Scarna17 | 2,74E+00 | 9,35E-03 |
| NM_175328 | Slc6a15 | 2,75E+00 | 9,09E-03 |
| ENSMUST00000135027 | Msn | 2,76E+00 | 8,82E-03 |
| NR_029880 | Mir379 | 2,76E+00 | 8,78E-03 |
| NR_015456 | D7Ertd715e | 2,78E+00 | 8,37E-03 |
| XR_141489 | L1Md-Gf21 | 2,78E+00 | 8,29E-03 |
| NM_009855 | Cd80 | 2,79E+00 | 8,06E-03 |
| NM_007643 | Cd36 | 2,80E+00 | 8,02E-03 |
| XM_001475753 | Ly6g | 2,80E+00 | 7,95E-03 |
| ENSMUST00000150745 | Nop56 | 2,81E+00 | 7,78E-03 |
| NM_027834 | 9130008F23Rik | 2,82E+00 | 7,54E-03 |
| NM_207246 | Rasgrp3 | 2,83E+00 | 7,25E-03 |
| NM_001173459 | LOC100038947 | 2,83E+00 | 7,22E-03 |
| ENSMUST00000079824 | Gpr84 | 2,84E+00 | 7,04E-03 |
| ENSMUST00000105105 | Hist2h3b Hist1h3e Hist1h3b Hist1h3d Hist1h3c Hist1h3f Hist2h3c2 Hist2h3c1 | 2,84E+00 | 7,03E-03 |
| ENSMUST00000090776 | Hist1h2ao Hist1h2ai Hist1h2ab Hist1h2ap Hist1h2an Hist1h2ah Hist1h2ag Hist1h2ae Hist1h2ad Hist1h2ac | 2,84E+00 | 7,02E-03 |
| ENSMUST00000067532 | Ms4a7 | 2,84E+00 | 6,99E-03 |
| NM_178695 | Prrg4 | 2,86E+00 | 6,66E-03 |
| NM_009448 | Tuba1c | 2,88E+00 | 6,40E-03 |
| ENSMUST00000040576 | Parm1 | 2,88E+00 | 6,39E-03 |
| NR_003564 | Gm15698 | 2,88E+00 | 6,30E-03 |
| X01134 | Trav9d-3 | 2,89E+00 | 6,08E-03 |
| NM_138648 | Olr1 | 2,90E+00 | 5,88E-03 |
| NR_029786 | Mir19a | 2,91E+00 | 5,82E-03 |
| NR_002455 | Snord34 | 2,91E+00 | 5,74E-03 |
| ENSMUST00000033910 | Leprotl1 | 2,92E+00 | 5,60E-03 |
| NR_029768 | Mir339 | 2,93E+00 | 5,53E-03 |
| NM_017469 | Gucy1b3 | 2,93E+00 | 5,47E-03 |
| NR_030718 | F630028O10Rik | 2,94E+00 | 5,33E-03 |
| NM_025961 | Gatm | 2,94E+00 | 5,26E-03 |
| NR_029564 | Mir154 | 2,95E+00 | 5,09E-03 |
| NM_001145801 | Ctla2b | 2,97E+00 | 4,85E-03 |
| AB010352 | AB010352 | 2,97E+00 | 4,83E-03 |
| NM_181444 | Gprc5a | 2,98E+00 | 4,64E-03 |
| ENSMUST00000068569 | Bcl2a1b | 3,06E+00 | 3,67E-03 |
| NM_016851 | Irf6 | 3,07E+00 | 3,60E-03 |
| NR_000002 | Snord32a | 3,08E+00 | 3,52E-03 |
| AK138760 | Gtf3c2 | 3,08E+00 | 3,43E-03 |
| NR_029555 | Mir142 | 3,09E+00 | 3,41E-03 |
| NM_009925 | Col10a1 | 3,09E+00 | 3,34E-03 |
| NR_030702 | Snord4a | 3,10E+00 | 3,31E-03 |
| NR_030570 | Mir466h | 3,14E+00 | 2,86E-03 |
| XR_107106 | Gm19389 | 3,20E+00 | 2,41E-03 |
| AK019796 | 4930570D08Rik | 3,23E+00 | 2,19E-03 |
| NM_011337 | Ccl3 | 3,23E+00 | 2,16E-03 |
| NM_031169 | Kcnmb1 | 3,28E+00 | 1,86E-03 |
| NM_175499 | Slitrk6 | 3,29E+00 | 1,79E-03 |
| ENSMUST00000152594 | Zbtb38 | 3,29E+00 | 1,76E-03 |
| NR_040692 | Gm10664 | 3,32E+00 | 1,59E-03 |
| NM_001005485 | Olfr111 | 3,34E+00 | 1,53E-03 |
| NM_001164044 | Ccl27a | 3,35E+00 | 1,44E-03 |
| AK145005 | 2010300F17Rik | 3,36E+00 | 1,42E-03 |
| NM_144862 | Lims2 | 3,37E+00 | 1,37E-03 |
| ENSMUST00000082059 | Erbb3 | 3,41E+00 | 1,21E-03 |
| NM_146406 | Olfr1076 | 3,44E+00 | 1,06E-03 |
| NM_023256 | Krt20 | 3,45E+00 | 1,04E-03 |
| NR_030540 | Mir882 | 3,45E+00 | 1,04E-03 |
| NR_035408 | Mir669a-1 | 3,46E+00 | 9,98E-04 |
| ENSMUST00000102968 | LOC100862646 Hist1h4m Hist1h4b Hist1h4a Hist4h4 Hist1h4n Hist1h4k Hist1h4j Hist1h4i Hist1h4f Hist1h4d Hist1h4c Hist2h4 Hist1h4h | 3,48E+00 | 9,49E-04 |
| NM_010373 | Gzme | 3,58E+00 | 6,46E-04 |
| NM_080450 | Gjc3 | 3,62E+00 | 5,60E-04 |
| NR_028554 | Snord70 | 3,65E+00 | 5,02E-04 |
| NM_009264 | Sprr1a | 3,68E+00 | 4,53E-04 |
| NR_028569 | Snord1c | 3,70E+00 | 4,23E-04 |
| NR_028569 | Snord1c | 3,78E+00 | 3,19E-04 |
| NM_013904 | Hey2 | 3,80E+00 | 2,95E-04 |
| NR_035500 | Mir1983 | 3,82E+00 | 2,67E-04 |
| ENSMUST00000110452 | Hist1h2bn Hist1h2bl Hist1h2bj Hist1h2bf | 3,85E+00 | 2,42E-04 |
| ENSMUST00000033800 | Plp1 | 3,85E+00 | 2,40E-04 |
| NR_030559 | Mir453 | 3,90E+00 | 1,96E-04 |
| NM_010867 | Myom1 | 3,92E+00 | 1,82E-04 |
| AK048657 | Lpin2 | 4,15E+00 | 7,41E-05 |
| NR_028528 | Snord57 | 4,16E+00 | 6,88E-05 |
| NR_045473 | 1810012K16Rik | 4,18E+00 | 6,53E-05 |
| NM_028749 | Npl | 4,28E+00 | 4,22E-05 |
| NR_029915 | Mir376b | 4,29E+00 | 4,08E-05 |
| NM_011332 | Ccl17 | 4,39E+00 | 2,58E-05 |
| ENSMUST00000091703 | Hist2h3b Hist1h3e Hist1h3b Hist1h3d Hist1h3c Hist1h3f Hist2h3c2 Hist2h3c1 | 4,49E+00 | 1,69E-05 |
| NM_010372 | Gzmd | 4,52E+00 | 1,44E-05 |
| NM_001081386 | Cdh19 | 4,58E+00 | 1,12E-05 |
| NR_029651 | Mir300 | 4,71E+00 | 5,94E-06 |
| NM_183391 | Tnfsf18 | 4,80E+00 | 4,06E-06 |
| NM_130866 | Olfr78 | 5,05E+00 | 1,14E-06 |
| NR_028551 | Snord53 | 5,56E+00 | 7,91E-08 |
| NR_029877 | Mir376a | 5,60E+00 | 6,02E-08 |
| ENSMUST00000102979 | LOC100862646 Hist1h4m Hist1h4b Hist1h4a Hist4h4 Hist1h4n Hist1h4k Hist1h4j Hist1h4i Hist1h4f Hist1h4d Hist1h4c Hist2h4 Hist1h4h | 5,73E+00 | 2,98E-08 |
| ENSMUST00000147599 | Rdh13 | 6,18E+00 | 2,02E-09 |
| NR_028567 | Snord1b | 6,26E+00 | 1,20E-09 |
| NM_025556 | 2410022L05Rik | 7,80E+00 | 2,41E-14 |
| NM_010818 | Cd200 | 8,84E+00 | 4,33E-18 |
